# The spitting image of plant defenses: Effects of plant secondary chemistry on the efficacy of caterpillar regurgitant as an anti‐predator defense

**DOI:** 10.1002/ece3.3174

**Published:** 2017-07-05

**Authors:** Gaylord A. Desurmont, Angela Köhler, Daniel Maag, Diane Laplanche, Hao Xu, Julien Baumann, Camille Demairé, Delphine Devenoges, Mara Glavan, Leslie Mann, Ted C. J. Turlings

**Affiliations:** ^1^ Institute of Biology University of Neuchâtel Neuchâtel Switzerland

**Keywords:** behavioral ecology, crucifers, defense against natural enemies, oral secretions, plant‐mediated effects, tritrophic interactions

## Abstract

In the arms race between plants, herbivores, and their natural enemies, specialized herbivores may use plant defenses for their own benefit, and variation in plant traits may affect the benefits that herbivores derive from these defenses. *Pieris brassicae* is a specialist herbivore of plants containing glucosinolates, a specific class of defensive secondary metabolites. Caterpillars of *P. brassicae* are known to actively spit on attacking natural enemies, including their main parasitoid, the braconid wasp *Cotesia glomerata*. Here, we tested the hypothesis that variation in the secondary metabolites of host plants affects the efficacy of caterpillar regurgitant as an anti‐predator defense. Using a total of 10 host plants with different glucosinolate profiles, we first studied natural regurgitation events of caterpillars on parasitoids. We then studied manual applications of water or regurgitant on parasitoids during parasitization events. Results from natural regurgitation events revealed that parasitoids spent more time grooming after attack when foraging on radish and nasturtium than on *Brassica* spp., and when the regurgitant came in contact with the wings rather than any other body part. Results from manual applications of regurgitant showed that all parameters of parasitoid behavior (initial attack duration, attack interruption, grooming time, and likelihood of a second attack) were more affected when regurgitant was applied rather than water. The proportion of parasitoids re‐attacking a caterpillar within 15 min was the lowest when regurgitant originated from radish‐fed caterpillars. However, we found no correlation between glucosinolate content and regurgitant effects, and parasitoid behavior was equally affected when regurgitant originated from a glucosinolate‐deficient *Arabidopsis thaliana* mutant line. In conclusion, host plant affects to a certain extent the efficacy of spit from *P. brassicae* caterpillars as a defense against parasitoids, but this is not due to glucosinolate content. The nature of the defensive compounds present in the spit remains to be determined, and the ecological relevance of this anti‐predator defense needs to be further evaluated in the field.

## 1 INTRODUCTION

The idea that plants affect not only their consumers, but also the interactions between the second and third trophic levels has received considerable attention since Price et al.'s seminal article in 1980. Plant traits that confer direct or indirect resistance against herbivores may have effects cascading up the food chain (Desurmont, Harvey et al., 2014; Hare, 2011; Harvey, Van Dam, & Gols, 2003). For example, the performance of parasitoids, which develop inside or on the outside of an herbivore host, has often been found to be correlated with the performance of the host: if plant defenses affect the host, parasitoid's performance is likely to be impacted the same way (Gols & Harvey, 2009; Gols et al., 2008). On the other hand, specialized herbivores may have adapted to their host plant's defenses and use them for their own benefit through the sequestration of plant toxins making them less palatable or better defended against natural enemies (Agrawal, Petschenka, Bingham, Weber, & Rasmann, 2012; Nishida, 2002; Opitz & Müller, 2009). In such cases, variation in plant defenses among host plants may directly impact the herbivore's level of defenses or unpalatability. For example, a host‐specialist aphid species known to sequester plant toxins was found to be less well defended against a generalist predator when feeding on plants with low levels of noxious chemicals (Francis, Lognay, Wathelet, & Haubruge, 2001). At an evolutionary level, the benefits that herbivores derive from plant defenses have the potential to drive host plant preferences, dietary specialization, and speciation processes for both herbivores and their host specialized natural enemies (Abrahamson & Blair, 2008; Ali & Agrawal, 2012; Quicke, 1997).

Studies on the effects of plant secondary chemistry on anti‐predator defenses have almost exclusively focused on sequestered toxins (Hartmann, 2004; Heckel, 2014), largely missing out on other strategies deployed by herbivores to fend off attackers (Karban & Agrawal, 2002). Moreover, most of these studies concern just a few host plants with a limited range of chemical profiles (Calcagno, Avila, Rudman, Otero, & Alonso‐Amelot, 2004; Francis et al., 2001; Sword, 2001). Here, we tested the hypothesis that plant chemistry affects the efficacy of another type of herbivore defense: the active regurgitation of oral secretions on natural enemies.

The secretion or regurgitation of defensive chemicals to fend off attackers has been documented in nature for a wide range of animals, including lizards (Middendorf & Sherbrooke, 1992) and birds (Canestrari et al., 2014; Warham, 1977). Among insects, examples of this defense mechanism include autohemorrhaging behavior (i.e., reflex bleeding) in several species of beetles and grasshoppers (Bateman & Fleming, 2009), secretion of deterrent chemicals from specialized glands (Eisner & Aneshansley, 1999; Pasteels, Rowell‐Rahier, & Raupp, 1988), and regurgitation of oral secretions and/or semi‐digested plant material (Hunter, 2000; Tullberg & Hunter, 1996). Defensive regurgitation is common among lepidopterans at the larval stage (Gross, 1993), and it is typically associated with an enlarged foregut in caterpillars using this weaponry (Grant, 2006). In the Pieridae family, the cabbage white *Pieris brassicae* is known to actively regurgitate on attacking natural enemies (Müller, Agerbirk, & Olsen, 2003), including its main parasitoid *Cotesia glomerata* (Hymenoptera: Braconidae) (Figure S1). Interestingly, closely related species such as *Pieris rapae* or *Pieris virginiensis* do not display such behavior (Hunter, 2000). Moreover, regurgitation was found to have an intrinsic cost for *P. brassicae* caterpillars, reducing pupal weight in caterpillars that regurgitated more frequently or more abundantly (Higginson, Delf, Ruxton, & Speed, 2011). These findings suggest that defensive regurgitation must have an adaptive value to be maintained in *P. brassicae* in nature. Müller et al. (2003) found that *P. brassicae* regurgitant is deterrent to ants, suggesting the presence of compounds with anti‐predator properties. Being a specialist feeder on glucosinolate‐containing plants, it is possible that *P. brassicae* derives its chemical weaponry from its host plants’ secondary metabolites.

Glucosinolates are a class of plant compounds known for their defensive function (Ahuja, Rohloff, & Bones, 2010; Hopkins, van Dam, & van Loon, 2009). There are more than 120 described glucosinolates, present in 16 plant families (Fahey, Zalcmann, & Talalay, 2001). Although they are known to be toxic or deterrent to many species of herbivores, several host‐specialized insect herbivores have adapted to deal with these compounds (Winde & Wittstock 2011). This is the case for several butterflies belonging to the *Pieris* genus, which use them as oviposition and/or feeding stimulants (Hopkins et al., 2009). Our study species, *P. brassicae*, develops mainly on wild and cultivated crucifers (family Brassicaceae), but is also known to infest other glucosinolate‐containing plants in nature, for example the ornamental nasturtium *Tropaeolum majus* (family Tropaeolaceae) (Geervliet et al., 2000).

To test the hypothesis that host plant secondary chemistry affects the efficacy of regurgitant as an anti‐predator defense in *P. brassicae*, and to investigate whether regurgitant efficacy is directly linked to glucosinolate concentration in plants, we conducted two experiments under laboratory conditions with young *P. brassicae* caterpillars and *C. glomerata* parasitoids. In the first experiment, the effect of regurgitation by caterpillars on parasitoids was directly observed on different host plants. In the second experiment, regurgitant collected from caterpillars reared on different host plants was manually applied on parasitoids attacking *P. brassicae* caterpillars. For both experiments, several parameters of the wasp behavior (described below) were recorded after contact with the regurgitant to evaluate its efficacy as a defense. We tested a total of 10 host plants to encompass a wide range of glucosinolate profiles. Specifically, we used three different populations of *Brassica rapa* with high, medium, and low glucosinolate content; three cultivated plants from the Brassicaceae family (*Brassica napus*,* Brassica oleracea*, and *Raphanus sativus*); two host plants not belonging to the Brassicaceae family (*T. majus* and *Cleome hassleriana*); and two genetic lines of *Arabidopsis thaliana* (Brassicaceae family): a genetically modified line deficient in glucosinolate production (*myc234* triple mutant) and the corresponding glucosinolate‐containing wild‐type (*Col‐0*) (Schweizer et al., 2013). To investigate the link between plant chemical profile and regurgitant efficacy, we extracted and measured the glucosinolate content in leaves of all host plants. Our working hypothesis was that *C. glomerata* behavior should be more strongly impacted by regurgitant originating from plants with higher glucosinolate concentrations, and we tested this prediction at three different levels: across all host plants, within the three *B. rapa* populations, and between the two *A. thaliana* lines.

## 2 MATERIALS AND METHODS

### 2.1 Plant and insect material

We used a total of 10 host plant species and varieties for the different experiments: two wild accessions of *B. rapa* (hereafter referred to as BR1 and BR2), originally collected in different locations of the Netherlands (Maarssen and Almere, respectively) and known for their high glucosinolate content (Tom de Jong, University of Leiden, personal communication), and one cultivated variety of the same species: the Chinese cabbage *B. rapa* var. *pekinensis* (BR3). We also used cultivated varieties of nasturtium *T. majus* (CAP), radish *R. sativus* (R), green cabbage *B. oleracea* (O), oilseed rape *B. napus* (BN), spider flower *C. hassleriana* (S), and two accessions of *A. thaliana*: a glucosinolate‐deficient mutant line that does not produce any glucosinolates (*myc234*) and the corresponding wild‐type, Columbia‐0 (*Col‐0*) (Schweizer et al., 2013). All plant species used belong to the Brassicaceae family, with the exception of *T. majus* (Tropaeolaceae) and *C. hassleriana* (Cleomaceae). All plants were grown in medium‐sized plastic pots (14 × 14 cm), except for *A. thaliana* plants, which were grown in smaller pots (4 × 5 cm). Plants were germinated and grown for the first 2 weeks in controlled phytotrons under a 16/8 L:D light regime at 25°C, light intensity 180–220 μmol m^−2^. They were then moved to a greenhouse until needed for the experiments (3–5 weeks after germination), except for *A. thaliana* plants, which were kept in controlled growth chambers under the same conditions of temperature and light intensity with a short day cycle to prevent them from flowering and were used 5 weeks after germination.

*Pieris brassicae* caterpillars used for the experiments came from a laboratory rearing originally started with individuals collected in the field in the Zürich area (Switzerland). The braconid parasitoid *C. glomerata* is the main natural enemy of *P. brassicae* in temperate Western Europe and attacks early larval instars of its host. Parasitoids used in this study came from a laboratory rearing originally started with individuals collected in the field in the Neuchâtel area (Switzerland). Parasitoids were reared on *P. brassicae* caterpillars from the laboratory rearing. Newly emerged parasitoids of both sexes were placed in Bugdorm‐1 cages (30 × 30 × 30 cm, Mega View Science Education Services Co. Ltd, Taiwan) at ambient temperature (ca. 25°) with water and honey for 48 h, a period that is typically sufficient to ensure successful mating. Then, the rearing cages were transferred in a growth chamber at 13°C (16/8 L:D light regime) with water and honey until parasitoids were needed for the experiments (1–3 weeks after emergence).

### 2.2 Observations of regurgitation events on different host plants

The aim of this experiment was to evaluate the efficacy of caterpillar regurgitation as an anti‐predator defense by observing the behavior of *C. glomerata* wasps attacking second instar *P. brassicae* caterpillars on different host plants. Specifically, we observed the first regurgitation event that occurred when a parasitoid attacked a caterpillar within the bioassay arena, and recorded the body part(s) touched by the regurgitation and the time spent grooming by the parasitoid after the attack (1 regurgitation event = 1 replicate). We used four treatments (host plants) for this experiment: *B. rapa* wild population 1 (BR1) (*N* = 20), *B. rapa* var. *pekinensis* (BR3) (*N* = 20), *T. majus* (CAP) (*N* = 19), and *R. sativus* (R) (*N* = 20). We categorized the body parts of *C. glomerata* wasps as follows: head, thorax, abdomen, legs, and wings. Grooming behavior was defined as parasitoid leg movements aimed at cleaning different body parts (Zhukovskaya, Yanagawa, & Forschler, 2013). We recorded the total duration of grooming until the parasitoid attacked a second caterpillar, or until the observation period was over (15 min after the initial attack). If a parasitoid interrupted grooming for more than a few seconds and resumed grooming afterwards, the durations of all grooming sequences were added to obtain the total grooming time. The behavior of an individual parasitoid was only recorded once (i.e., one parasitoid was used per replicate). Caterpillars used for this experiment were feeding in groups of various sizes (10–25 caterpillars per group) and had been developing on their host plant since egg hatch. The number of regurgitation events that were recorded per group of caterpillars varied depending on the host plant and availability of caterpillars.

### 2.3 Manual application of regurgitant on *Cotesia glomerata* parasitoids

We collected regurgitant from *P. brassicae* caterpillars feeding on leaves of 10 host plant species (listed above). Regurgitant was obtained by holding the caterpillar between two fingers and gently applying pressure below the head, while collecting the regurgitant directly from the mouthparts using a micropipette. The regurgitant was immediately stored on ice, pooled for each plant species, and frozen at −20°C until use. Regurgitant was collected from third to fifth instar larvae, except for the two *A. thaliana* lines for which second and third instar larvae were used because these plants did not produce enough foliage to sustain bigger caterpillars. For the bioassay, we placed a leaf circle of *B. rapa* var. *pekinensis* with groups of newly hatched first instar *P. brassicae* on moist filter paper into a Petri dish. Newly hatched larvae were chosen for this experiment because they are too small to produce significant amounts of regurgitant and thus should not interfere with the manual application of regurgitant. Using an aspirator, we transferred individual *C. glomerata* females, to the Petri dish and placed them in close proximity to the feeding larvae. We applied 2.5 μl regurgitant with a micropipette onto the thorax once the wasp had inserted its ovipositor into a larva. An application of 2.5 μl water was used as control treatment. We then recorded the behavior of the parasitoid until it attacked a second larva (re‐attack), or for a maximum observation time of 15 min. The following parameters were recorded: attack interruption upon regurgitant application (yes/no), initial attack duration (s), grooming time (s), re‐attack within 15 min after the initial attack (yes/no), and time until re‐attack after regurgitant application (s). As before, all grooming sequences were added to obtain the total grooming time. Because all plant species could not be grown at the same time, we conducted three separate series of this experiment with different parasitoids, larval batches, and host plants tested. A total of 332 manual applications of *P. brassicae* regurgitant from 10 host plants on *C. glomerata* parasitoids were performed across the three experimental series (series 1: *N* = 92; series 2: *N* = 144; series 3: *N* = 96). Regurgitant collected on three host plants (BR1 = *B. rapa rapa* wild population 1, BR3 = *B. rapa* var. *pekinensis*, R = *R. sativus*) and water (control treatment) were tested in all three experimental series (BR1: *N* = 44; BR3: *N* = 49; R: *N* = 42; Water: *N* = 59). The details of the host plants tested for each experimental series are given in Table 1.

**Table 1:** Parasitoid behavior after manual exposure to caterpillar regurgitant

Series	Host plant	*N*	Attack interruption (%)	Attack duration (s)	Grooming duration (s)	Re‐attack (%)	Time of re‐attack (s)
				*F*_5,83_ = 1.1*p* = .35	***F***_**5,83**_ ** = 3.5** ***p*** ** < .01**	***df*** ** = 5 χ** ^**2**^ ** = 23.7** ***p*** ** < .0001**	***F***_**5,83**_ ** = 4.2** ***p*** ** < .01**
1	BR1	15		16.5 ± 2.7	466.7 ± 92.7a	46.7b	563.8 ± 99.7a
1	BR2	16		19.6 ± 4.0	364.4 ± 102.1ab	56.3b	457.6 ± 106.7ab
1	BR3	15		20.6 ± 3.8	316.7 ± 88.4ab	80.0b	401.4 ± 104.0ab
1	CAP	15		27.4 ± 5.2	445.4 ± 100.2a	40.0b	625.6 ± 96.6a
1	R	16		13.9 ± 2.7	538.9 ± 85.9a	25.0c	710.1 ± 87.9a
1	Water	15		25.9 ± 8.2	75.8 ± 32.5b	100.0a	150.7 ± 35.2b
			*df* = 8 χ^2^ = 7.1*p* = .5	*F*_8,135_ = 1.0*p* = .46	***F***_**8,135**_ ** = 2.4** ***p*** ** = .02**	***df*** ** = 8 χ** ^**2**^ ** = 10.5** ***p*** ** = .2**	***F***_**8,135**_ ** = 2.4** ***p*** ** = .02**
2	BR1	14	28.6	15.1 ± 3.5	324.2 ± 103.5ab	57.1	417.7 ± 116.2ab
2	BR2	16	18.8	19.1 ± 6.3	503.8 ± 93.5a	50.0	586.8 ± 96.3a
2	BR3	14	35.7	16.1 ± 4.2	374.1 ± 98.8ab	57.1	465.6 ± 113.2ab
2	CAP	17	5.9	26.5 ± 8.8	397.1 ± 83.2ab	64.7	512.7 ± 94.2ab
2	R	18	33.3	9.7 ± 2.0	418.0 ± 88.7ab	55.6	477.7 ± 96.4ab
2	O	13	30.8	16.4 ± 4.2	360.8 ± 102.8ab	61.5	404.9 ± 114.3ab
2	BN	15	33.3	17.9 ± 9.6	238.0 ± 77.9ab	73.3	382.5 ± 106.9ab
2	S	13	23.1	12.3 ± 3.4	438.5 ± 100.5ab	46.2	613.4 ± 109.6a
2	Water	24	16.7	12.1 ± 1.5	111.8 ± 47.5b	87.5	167.9 ± 60.1b
			*df* = 5 χ^2^ = 13.5*p* = .02	*F*_5,89_ = 2.5*p* = .04	***F***_**5,89**_ ** = 4.6** ***p*** ** = .001**	*df* = 5 χ^2^ = 5.5*p* = .3	***F***_**5,89**_ ** = 3.2** ***p*** ** = .01**
3	BR1	15	26.7a	8.8 ± 2.3b	30.9 ± 10.9bc	100.0	52.4 ± 27.0ab
3	BR3	20	30.0a	13.5 ± 3.6ab	134.8 ± 36.2a	95.0	153.2 ± 46.5a
3	R	8	37.5a	11.1 ± 3.5ab	70.3 ± 37.0abc	87.5	44.5 ± 17.4ab
3	Col‐0	19	15.8a	16.4 ± 2.3ab	84.0 ± 24.0ab	100.0	129.8 ± 34.5ab
3	*myc234*	14	50.0a	9.9 ± 2.9ab	67.6 ± 23.3abc	92.9	118.2 ± 42.0ab
3	Water	20	0.0b	23.7 ± 5.4a	13.4 ± 4.0c	100.0	39.0 ± 6.0b

2.5 μl regurgitant from *Pieris brassicae* caterpillars fed on 10 host plant species was applied manually onto the thorax of *Cotesia glomerata* females upon caterpillar attack. Parasitoid behavior was recorded until it re‐attacked another caterpillar, or for a maximum of 15 min. Data are shown for the three experimental series (proportions and means ± SE). BR1 = *Brassica rapa rapa* wild population 1, BR2 = *Brassica rapa rapa* wild population 2, BR3 = Chinese cabbage *Brassica rapa* var. *pekiniensis*, CAP = nasturtium *Tropaeolum majus*, R = radish *Raphanus stivus*, O = green cabbage *B. oleracea*, BN = oilseed rape *Brassica napus*, S = spider flower *Cleome hassleriana*,* Col‐0* = *Arabidopsis thaliana* wild‐type, *myc234 *= *Arabidopsis thaliana* glucosinolate‐free mutant, water = control treatment. Within each parameter and series, means followed by a different letters are statistically different (*p* < .05, ANOVAs for discrete variables, Chi‐square tests for proportions). Bold values indicate significant defferences.

### 2.4 Glucosinolate extraction and analysis

To examine the glucosinolate profiles of the different host plants following herbivory, five individuals of each host plant were infested with 30 L1 *P. brassicae* caterpillars, except for *Arabidopsis* plants for which only 15 L1 caterpillars were used due to their smaller size. Plants were kept in inverted PET bottles (9 × 28 cm) from which the bottom had been cut out. The opening was covered with a fine mesh to prevent the insects from escaping. After 24 h of feeding, all caterpillars were carefully removed and one fully expanded damaged leaf per plant was excised, immediately snap‐frozen in liquid nitrogen and stored at −80°C until sample preparation. We carefully selected leaves that had experienced comparable amounts of damage (visual estimation).

Glucosinolate profiles were analyzed following a modified protocol from Glauser, Schweizer, Turlings, and Reymond (2012). In brief, 11.5 (±1.5) mg of frozen leaf powder were suspended in 1 ml of cold methanol/water (70:29.5 v/v; 0.5% formic acid) and five glass beads were added. In contrast to Glauser et al. (2012), we used acidified extraction solvent to quench residual enzyme activity without any detectable influence on the analysis’ outcome compared to heat inactivation. Following a brief vortex and agitation in a bead mill at 30 s^−1^ for 3 min, all samples were centrifuged at 14 000 rpm for 5 min (20 800× *g*). Subsequently, 500 μl of the supernatants was transferred to glass vials and stored at −80°C until analysis. Glucosinolates were quantitated using a Waters Acquity UPLC^™^ system equipped with an Acquity charged surface hybrid (CSH) C_18_ column (Waters, 100 × 2.1 mm i.d., 1.7 μm particle size) that was connected to a Synapt G2 QTOF mass spectrometer (Waters, Milford, MA, USA) (Glauser et al., 2012). Gluconapin was used at 0.2, 1, 5, and 20 μg/ml to establish a calibration curve for glucosinolate quantitation except for the two *A. thaliana* accessions where glucobrassicin and glucoraphanin were used at a single concentration (2.5 μg/ml) to estimate glucosinolate contents.

### 2.5 Statistical analysis

For the first experiment, parasitoid grooming time was analyzed by running a two‐way ANOVA with host plant, body part reached, and the interaction between these two terms as effects in the model. Data were square root transformed to meet the assumptions of the model. Means were compared using a Tukey–Kramer post‐hoc test. An additional analysis was conducted to determine whether grooming time differed when more than one body part was reached. For this analysis, data from all observations where one body part was reached were pooled and were compared to observations where more than one body part was reached using a one‐way ANOVA. For the second experiment, data for the treatments that were common among the three series of the experiment (BR1, BR3, R, and water) were pooled together and the effects of host plant, series, and the interaction between the two terms on the parameters recorded (attack interruption upon regurgitant application (yes/no), initial attack duration (s), grooming time (s), re‐attack within 15 min after the initial attack (yes/no), and time until re‐attack (s)) were analyzed. In addition, data were also analyzed separately for each series, testing the effect of host plant on the parameters recorded. For these analyses, general ANOVAs for continuous variables and Chi‐square tests for proportion variables were used. Means were compared using a Tukey–Kramer post‐hoc test, and data were square root transformed to meet the assumptions of the model if necessary. Total glucosinolate levels were calculated for each plant by summing the concentrations of individual compounds. Genotypic effects on total glucosinolates were then analyzed using a one‐way ANOVA followed by a Holm–Sidak post‐hoc test. Because the glucosinolate extraction and quantification protocol were not done at the same time for the two *A. thaliana* accessions and the other host plant tested, they were excluded from this analysis. All data were square root transformed prior to the analysis. Associations between glucosinolate content (total levels and individual compounds) and parameters of parasitoid behavior (attack duration, grooming time, and time to re‐attack) were investigated using linear regression procedures with a Bonferroni correction (JMP9).

## 3 RESULTS

### 3.1 Observations of regurgitation events on different host plants

**Figure 1:**
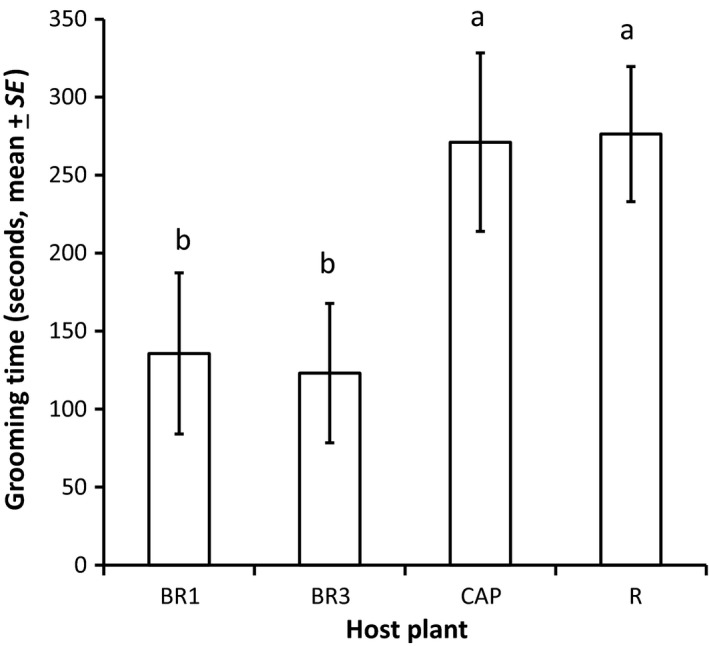
Time (seconds, mean ± SE) spent grooming by *Cotesia glomerata* parasitoids reached by caterpillar regurgitant on different host plants. BR1, *Brassica rapa rapa* wild population 1; BR3, Chinese cabbage *B. rapa* var. *pekinensis*; CAP, nasturtium *Tropaeolum majus*; R, radish *Raphanus sativus*. Treatments followed by a different letter are statistically different (α = 0.05, one‐way ANOVA, JMP9) (*N* = 19 or 20 for each species)

**Figure 2:**
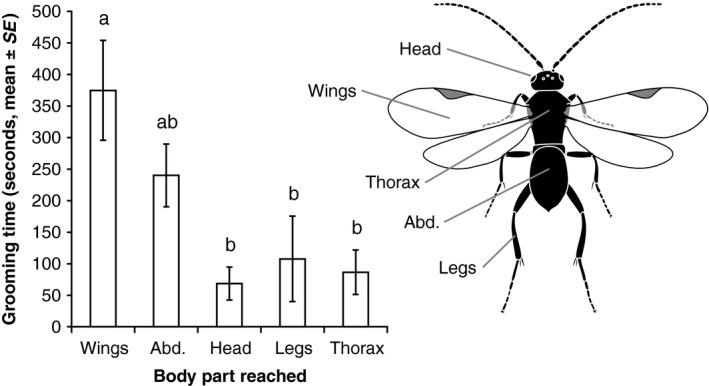
Time (seconds, mean ± SE) spent grooming by *Cotesia glomerata* wasps when reached by caterpillar regurgitant on different body parts: wings (*N* = 13), abdomen (i.e., metasoma) (*N* = 19), head (*N* = 8), legs (*N* = 7), and thorax (i.e., mesosoma) (*N* = 6). Illustration by Yves Borcard and Thomas Degen. Treatments followed by a different letter are statistically different (α = 0.05, one‐way ANOVA, JMP9)

Out of 80 parasitoid attacks followed by caterpillar regurgitation observed on the four host plants used in this experiment, 52 regurgitation events reached one body part, 20 reached multiple body parts, and in eight cases the body part(s) reached could not be determined with certainty. In cases where the regurgitant reached one body part, the abdomen was reached 34.6% of the time (18 observations), wings 25.0% (13 observations), head 15.4% (eight observations), legs 13.4% (seven observations), and thorax 11.5% (six observations). Variation in grooming time after regurgitation was significantly explained by the variables included in the model (*F*_8,63_ = 3.7, *p* = .001, *R* ^2^ = .23). Host plant species (*F*_3,63_ = 3.4, *p* = .02) and body part reached (*F*_5,63_ = 2.6, *p* = .03) both had a significant effect on grooming time, but not interaction between the two terms (*p* > .05). Parasitoids spent more time grooming when on radish (R) and nasturtium (CAP) than on *B. rapa* accessions (BR1 and BR3) (Figure 1). In observations where only one body part was reached, we found that parasitoids spent the most time grooming when their wings had been fouled; grooming time was intermediate when the abdomen was reached, and lower when the head, legs, or thorax were reached (Figure 2). Grooming was not significantly higher when multiple body parts were reached (186.7 ± 41.7 s) than when one body part was reached (211.9 ± 31.9) (*F*_1,70_ = 0.13, *p* = .71).

### 3.2 Manual application of regurgitant on *Cotesia glomerata* parasitoids

**Figure 3:**
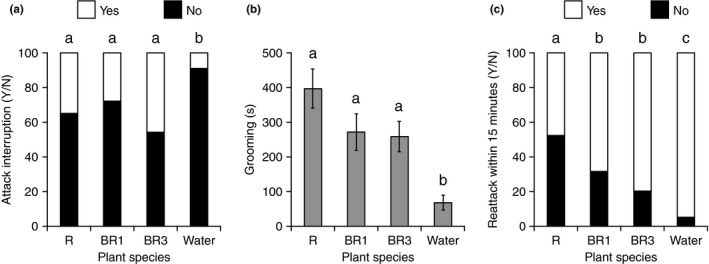
Effects of manual application of *Pieris brassicae* regurgitant from different host plants on *Cotesia glomerata* during attacks on caterpillars. (a) Proportion of attacks interrupted (Y/N) after application of regurgitant, (b) grooming time (seconds, mean ± SE), (c) proportion of attack of a second caterpillar within 15 minutes (Y/N) after application of the regurgitant. Host plant treatments are as follows: BR1, *Brassica rapa rapa* wild population 1 (*N* = 44); BR3, *B. rapa* var. *pekinensis* (*N* = 49); R, *Raphanus sativus* (*N* = 42), and water, control treatment where water was applied on parasitoids (*N* = 59). Treatments followed by a different letter are statistically different [α = 0.05, (a) and (c) Chi‐square test of proportions, (b) Two‐way ANOVA, JMP9]

The analysis of the pooled data of the four treatments common to the three experimental series (BR1, BR3, R, and water) overall revealed that the behavior of parasitoids was less affected when water was applied rather than regurgitant and that regurgitant originating for radish‐fed caterpillars (R) had a stronger impact on parasitoid behavior (significantly lower proportion of re‐attacks within 15 min and highest grooming time) than regurgitant originating from other host plants (Figure 3). In detail, the variables included in the model (host plant, series, and interaction between both terms) did not explain variation in parasitoid attack duration (*F*_7,125_ = 1.6, *p* = .14), but they had a significant effect on the proportion of attacks interrupted (*df* = 7, χ^2^ = 14.8, *p* = .04), on grooming time (*F*_11,182_ = 7.1, *p* < .0001), on the proportion of observing a re‐attack within 15 min (*df* = 11, χ^2^ = 68.3, *p* < .0001), and on the timing to re‐attack (*F*_11,182_ = 8.6, *p* < .0001). Host plant (*df* = 3, χ^2^ = 13.5, *p* < .01) had a significant effect on the proportion of attacks interrupted, but not series (*df* = 1, χ^2^ = 2.9, *p* = .08), nor the interaction between both terms (*df* = 3, χ^2^ = 4.5, *p* = .2). More parasitoid attacks were interrupted with regurgitant from BR1, BR3, and R, than with water (Figure 3a). Host plant (*F*_3,182_ = 11.0, *p* < .0001) and series (*F*_2,182_ = 15.1, *p* < .0001) had a significant effect on grooming time, but not the interaction between both terms (*F*_6,182_ = 1.8, *p* = .1): grooming time was longer with BR1, BR3, and R than with water (Figure 3b). Host plant (*df* = 3, χ^2^ = 15.7, *p* = .001) and series (*df* = 2, χ^2^ = 22.1, *p* < .0001) had a significant effect on the proportion of observing a re‐attack within 15 min, but not the interaction between both terms (*df* = 6, χ^2^ = 10.7, *p* = .09). Proportion of second attacks was the lowest with R, higher with BR1 and BR3, and the highest with water (Figure 3c). Host plant (*F*_3,182_ = 7.4, *p* = .0001) and series (*F*_2,182_ = 27.1, *p* < .0001), and the interaction between both terms (*F*_6,182_ = 2.4, *p* = .02) had a significant effect on time to re‐attack. For series 1, time to re‐attack was higher with R and BR1, intermediate with BR3, and lower with water (*F*_3,57_ = 6.3, *p* < .001). For series 2, time to re‐attack was higher with R, intermediate with BR1 and BR3, and lower with water (*F*_3,66_ = 3.2, *p* = .01). For series 3, time of re‐attack was higher with BR3 and lower with all other treatments (*F*_3,59_ = 4.3, *p* = .01). The significant effects of series on parasitoid behavior followed the same pattern for grooming time, proportion of observing a re‐attack within 15 min, and time to re‐attack: parasitoid behavior was similarly affected during series 1 and 2, but drastically less affected (i.e., lower grooming time, higher proportion of re‐attacking a caterpillar, and shorter time to re‐attack) during series 3 (Fig. S1).

The additional analyses of the three experimental series separately further supported that applying water to the parasitoids had much less impact on their behavior than the application of regurgitant (Table 1). However, we found very few significant differences among host plants within each series. Specifically, in series 1, proportion of re‐attacks within 15 min was lower on R than on all other host plants; in series 3, grooming duration was higher on BR3 than on BR1 (Table 1). Importantly, we did not find any difference in parasitoid behavior when exposed to regurgitant originating from an *Arabidopsis* line containing glucosinolates or an *Arabidopsis* line deficient in glucosinolates (Table 1, series 3).

### 3.3 Glucosinolate analysis

**Figure 4:**
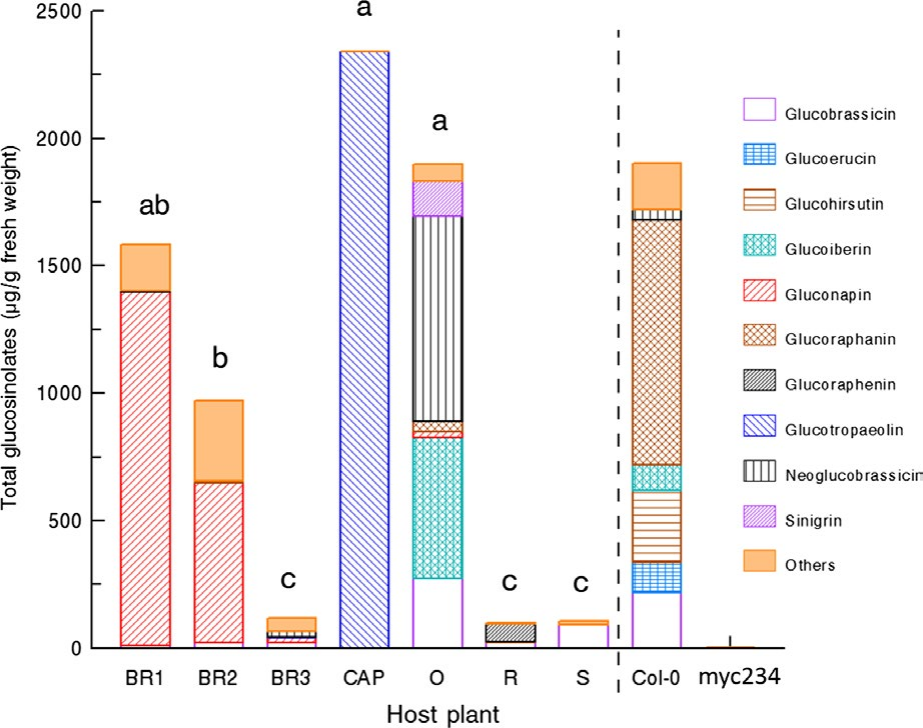
Leaf glucosinolate profiles following 24 h of *Pieris brassicae* caterpillar feeding (μg/g fresh weight; means ± SE;* N* = 5, except *Col‐0*:* N* = 4). BR1, *Brassica rapa rapa* wild population 1; BR2, *Brassica rapa rapa* wild population 2; BR3, Chinese cabbage *Brassica rapa* var. *pekinensis*; CAP, nasturtium *Tropaeolum majus*; R, radish *Raphanus sativus*; O, green cabbage *Brassica oleracea*; S, spider flower *Cleome hassleriana*;* Col‐0*,* Arabidopsis thaliana* wild‐type; *myc234*,* A. thaliana* glucosinolate‐free mutant. Means followed by a different letter indicate that the total glucosinolate quantity is statistically different between two host plants (one‐way ANOVA, α = 0.05, JMP9). Glucosinolate quantification for *A. thaliana* accessions was done at a later time than the other host plants and they were excluded from the analysis; the dashed line indicates this separation

Following infestation by *P. brassicae*, we detected 20 different glucosinolates across the nine host plants that were tested, and the different host plants differed remarkably in their glucosinolate profiles both in quantity and quality (Figure 4, Table S1). As expected, we found only trace amounts of glucosinolates in the *myc234* triple mutant of *A. thaliana* (<1 μg/g FW). By contrast, the wild‐type, Col‐0, contained nearly 2000 μg/g FW of total glucosinolates. Among the remaining seven host plants, the highest total amounts were detected in CAP (2341 μg/g FW), while R displayed the lowest glucosinolate levels (100 μg/g FW) with the predominant compounds by far being glucotropaeolin and glucoraphenin, respectively. The three *B. rapa* accessions had slightly more complex glucosinolate blends (Table S1). The quantitative variation among the three accessions, however, was similarly high ranging from 1583 μg/g FW for BR1 to 117 μg/g FW for BR3. BR2 showed intermediate glucosinolate levels. Overall, host plant explained 87% of the observed quantitative variation in glucosinolate content (*F*_6,28_ = 32.462, *p* < .001). We found no significant associations between total glucosinolate levels or the concentrations of individual glucosinolates and parasitoid behavior parameters (attack duration, grooming time, time to re‐attack) (*Ps* > .05, JMP9).

## 4 DISCUSSION

### 4.1 Regurgitation as a defense against natural enemies

Overall, we found good evidence of the efficacy of regurgitant as a defense, with parasitoids more likely to interrupt their attack, spending more time grooming and less likely to re‐attack a caterpillar within 15 min after exposure to regurgitant than after exposure to water (Figure 3). Regurgitant was observed to stick to the hydrophobic cuticle of the parasitoid, whereas droplets of water were simply deflected. This confirms that *P. brassicae* regurgitant is highly amphiphilic and acts as a surfactant that can wet the cuticle of arthropod attackers, as it is the case for regurgitant of other caterpillars (Rostás & Blassmann, 2009). Moreover, grooming time was dependent on the body part that was covered with the regurgitant, with wings being the most time‐consuming body part to groom (Figure 2). We also found direct evidence that host plant influences to a certain extent the efficacy of regurgitation. Indeed, parasitoid behavior was more affected when regurgitant originated from radish than from wild or cultivated *B. rapa* plants: grooming time was longer during observations of natural regurgitation events (Figure 1) and the likelihood of re‐attacking caterpillars was lower in the regurgitant application experiment (Figure 3). Nasturtium also elicited a longer grooming time than *B. rapa* plants during observations of natural regurgitation events (Figure 1).

### 4.2 Host plant glucosinolate profile and regurgitant efficacy

Overall, we found very little support for the hypothesis that plant glucosinolate content is associated with efficacy of *P. brassicae* regurgitant. There were no significant correlations between total glucosinolate levels or quantities of single glucosinolate compounds and parasitoid behavior for any of the response parameters tested, and parasitoid behavior was not differently affected when parasitoids were exposed to regurgitant originating from *B. rapa* populations with high, medium, and low glucosinolate content (BR1, BR2, and BR3, respectively). Moreover, the fact that regurgitant was not less effective when originating from a glucosinolate‐deficient *A. thaliana* accession rules out an important role for glucosinolates in anti‐parasitoid defense. Still, it should be noted that the host plant that had the strongest effect on the parasitoids was *R. sativus* (R), a host plant with a unique glucosinolate profile, albeit in low concentrations. Indeed, the major glucosinolate present in *R. sativus*, glucoraphenin, is absent in the other host plants tested (Table S1).

It appears that the anti‐predator properties of *P. brassicae* regurgitant may be based on self‐synthetized compounds or on plant‐derived compounds other than glucosinolates. Most of the research on plant defenses in the Brassicaceae family has focused on the role of glucosinolates as major mediators of insect–plant interactions (Hopkins et al., 2009), but non‐glucosinolate key compounds have also been documented (Roessingh, Städler, Baur, Hurter, & Ramp, 1997). Testing the spit of caterpillars reared on artificial diet would be one possible way to further elucidate the effects of host plant chemistry on regurgitant efficacy, but is challenging to do with *P. brassicae*.

### 4.3 Significance of this research and future directions

Plant‐mediated effects of defensive regurgitation in insects have been studied in orthopterans (Calcagno et al., 2004; Sword, 2001), but have rarely been examined for lepidopteran larvae (but see Higginson et al., 2011). Our study is a first attempt to unravel the link between plant chemistry and anti‐predator defenses in *P. brassicae*, a non‐sequestering herbivore. The two experimental designs that we used had different advantages and limitations that may have affected the results. On the one hand, direct observations of regurgitation events on different host plants allowed us to observe the anti‐predator proprieties of regurgitant in a realistic context. However, observations on different host plants make the effects of regurgitant chemistry impossible to distinguish from other plant traits that could potentially have played a role on parasitoid behavior. For example, parasitoids may have been more motivated to look for hosts on the familiar *B. rapa* plants rather than on radish and nasturtium, prompting a shortened grooming time (Figure 1). Moreover, observations of natural regurgitation behavior did not allow us to control for the volume of regurgitant expelled by caterpillars, or other parameters of caterpillar performance.

In contrast, manual applications of regurgitant on parasitoids during attacks on first instar caterpillars allowed us to standardize host plant traits in the bioassay arena (leaf disks of Chinese cabbage) and the volume of regurgitant applied on caterpillars, but the process of collecting and storing it prior to the experiments may have caused the regurgitant to lose some of its properties. In their studies on grasshoppers, Sword (2001) and Calcagno et al. (2004) used similar collecting and storing techniques, but the specificities of our system may have produced a different outcome. A detailed investigation of the chemical composition of *P. brassicae*'s regurgitant and different handling and storing techniques would be needed to explore some of these possibilities.

In conclusion, our study supports the idea that the value of *P. brassicae*'s regurgitant as an anti‐predator defense is mainly due to its amphiphilic proprieties (Rostás & Blassmann, 2009), with little effect of plant secondary metabolites on the deterrence of the regurgitant. Ecologically, possessing an anti‐predator defense whose efficacy is independent of plant chemistry may be beneficial for *P. brassicae*, by alleviating some possible costs of host plant switching and diet mixing in nature. Given that amphiphilic properties have been reported for a wide range of caterpillar regurgitants (Rostás & Blassmann, 2009), it is therefore puzzling that close relatives of *P. brassicae*,* P. rapae*, and *P. virginiensis* do not actively regurgitate on natural enemies (Hunter, 2000). This may have something to do with the gregarious feeding behavior of *P. brassicae*, which is also distinctive among pierids. Young *P. brassicae* larvae feed in close groups up to the third instar, whereas its relatives are typically solitary. The benefits of defensive regurgitation for *P. brassicae* may only be present or enhanced in the context of gregarious feeding. In our study, regurgitation rarely prevented caterpillars from getting stung by parasitoids, as only a few attacks were interrupted after exposure to regurgitant (Table 1). Yet, successful regurgitation on a parasitoid by a caterpillar within a feeding group may help protect its kin within the group, and thus regurgitation may be more effective for gregarious species than solitary species. Indeed, each exposure to regurgitant made parasitoids waste time and energy grooming, which may benefit members of a group by reducing the overall attack rate (Turchin & Kareiva, 1989). Moreover, repeated exposures to deterrent chemicals may have more serious effects on parasitoids than one‐time exposures. Consequently, the adaptive value of defensive regurgitation for *P. brassicae* should be investigated in the field in the context of its gregarious behavior, without underestimating the possible effects of host plant traits on the benefits of group living (Desurmont, Weston & Agrawal 2014).

## DATA ARCHIVING

The authors are willing to make the dataset used in this study fully available on the DRYAD online database upon publication of this paper.

## CONFLICT OF INTEREST

None declared.

## AUTHORS' CONTRIBUTIONS

All authors contributed to the design of the study. All authors but TCJT collected the data; GAD and DM analyzed the data; GAD, DM, and AK wrote the initial draft of the manuscript; and all authors contributed to subsequent versions of the manuscript.
