# Supplementary material for: The spitting image of plant defenses: Effects of plant secondary chemistry on the efficacy of caterpillar regurgitant as an anti‐predator defense
Source: Ecol Evol. 2017 Jul 5;7(16):6304–13. doi: 10.1002/ece3.3174 (PMC5574803; doi:10.1002/ece3.3174)
Supplement: Supplementary file 1 [file ECE3-7-6304-s001.docx]

**Table S1. Glucosinolate profiles of the host plants.** Leaf glucosinolate profiles following 24 h of *P. brassicae* caterpillar feeding (all concentrations are µg / g fresh weight; means ± SE; N=5, except Col-0: N=4). BR1 = *Brassica rapa rapa* wild population 1, BR2 = *Brassica rapa rapa* wild population 2, BR3 = Chinese cabbage *Brassica rapa* var. *pekiniensis*, CAP = nasturtium *Tropaeolum majus*, R = radish *Raphanus sativus*, O = green cabbage *B. oleracea*, S = spider flower *Cleome hassleriana*, Col-0 = *Arabidopsis thaliana* wild type, *myc234* = *Arabidopsis thaliana* glucosinolate-free mutant. n.d. = not detected.

| Compound | BR1 | BR2 | BR3 | CAP | R | O | S | Col-0 | *myc234* |
| --- | --- | --- | --- | --- | --- | --- | --- | --- | --- |
| Gluconapin | 1384.8 ± 234.4 | 627.7 ± 152.2 | 19.5 ± 18.7 | n.d. | n.d. | n.d. | n.d. | n.d. | n.d. |
| Progoitrin | 57.9 ± 30.9 | 82.4 ± 50.0 | 22.8 ± 19.4 | n.d. | n.d. | 20.1 ± 3.7 | n.d. | n.d. | n.d. |
| Butyl-GS | 44.2 ± 25.4 | 161.6 ± 79.3 | n.d. | n.d. | n.d. | 2.1 ± 0.5 | 6.7 ± 4.4 | n.d. | n.d. |
| Glucobrassicanapin | 59.2 ± 11.9 | 59.8 ± 21.3 | 19.4 ± 16.4 | n.d. | n.d. | n.d. | n.d. | n.d. | n.d. |
| Glucotropaeolin | 0.8 ± 0.6 | 0.1 ± 0.1 | 2.5 ± 1.1 | 2340.3 ± 300.5 | n.d. | n.d. | n.d. | n.d. | n.d. |
| Glucobrassicin | 11.9 ± 2.5 | 21.5 ± 11.3 | 22.0 ± 6.1 | n.d. | 21.9 ± 8.0 | 274.5 ± 64.4 | 90.5 ± 17.2 | 217.2 ± 14.3 | 0.2 ± 0.04 |
| Hydroxyglucobrassicin | 2.0 ± 0.5 | 3.5 ± 2.0 | 3.8 ± 1.1 | n.d. | 3.3 ± 1.3 | 35.9 ± 7.9 | 9.2 ± 1.6 | 14.1 ± 1.5 | 0.1 ± 0.04 |
| Gluconasturtiin | 17.0 ± 7.5 | 5.8 ± 1.7 | 3.5 ± 1.9 | n.d. | n.d. | 2.1 ± 0.6 | n.d. | n.d. | n.d. |
| Glucoraphanin | n.d. | 5.1 ± 3.4 | n.d. | 0.3 ± 0.3 | 1.8 ± 1.2 | 39.9 ± 7.1 | n.d. | 960.9 ± 71.3 | 0.7 ± 0.1 |
| Glucoraphenin | n.d. | n.d. | n.d. | n.d. | 73.2 ± 41.5 | n.d. | n.d. | n.d. | n.d. |
| Glucoiberin | n.d. | n.d. | n.d. | n.d. | n.d. | 553.7 ± 80.4 | n.d. | 101.3 ± 7.5 | 0.1 ± 0.03 |
| Sinigrin | 0.8 ± 0.3 | 0.8 ± 0.2 | 0.1 ± 0.1 | n.d. | n.d. | 136.4 ± 30.9 | n.d. | n.d. | n.d. |
| Methoxyglucobrassicin | 2.4 ± 1.0 | 1.6 ± 0.5 | 2.8 ± 0.7 | n.d. | n.d. | 28.7 ± 4.1 | n.d. | 14.6 ± 0.9 | 0.6 ± 0.2 |
| Neoglucobrassicin | 2.2 ± 0.4 | 1.5 ± 0.7 | 20.7 ± 7.8 | n.d. | n.d. | 806.2 ± 172.5 | 0.8 ± 0.5 | 37.5 ± 14.1 | 0.1 ± 0.04 |
| Glucoibarin | n.d. | n.d. | n.d. | n.d. | n.d. | n.d. | n.d. | 46.7 ± 5.6 | 0.1 ± 0.02 |
| Glucoalyssin | n.d. | n.d. | n.d. | n.d. | n.d. | n.d. | n.d. | 33.6 ± 4.3 | n.d. |
| Glucohirsutin | n.d. | n.d. | n.d. | n.d. | n.d. | n.d. | n.d. | 281.3 ± 19.7 | 0.1 ± 0.03 |
| Glucoerucin | n.d. | n.d. | n.d. | n.d. | n.d. | n.d. | n.d. | 120.9 ± 12.7 | 0.1 ± 0.1 |
| 7-Methylthioheptyl-GS | n.d. | n.d. | n.d. | n.d. | n.d. | n.d. | n.d. | 17.2 ± 1.6 | n.d. |
| 8-Methylthiooctyl-GS | n.d. | n.d. | n.d. | n.d. | n.d. | n.d. | n.d. | 58.7 ± 4.7 | 0.1 ± 0.04 |


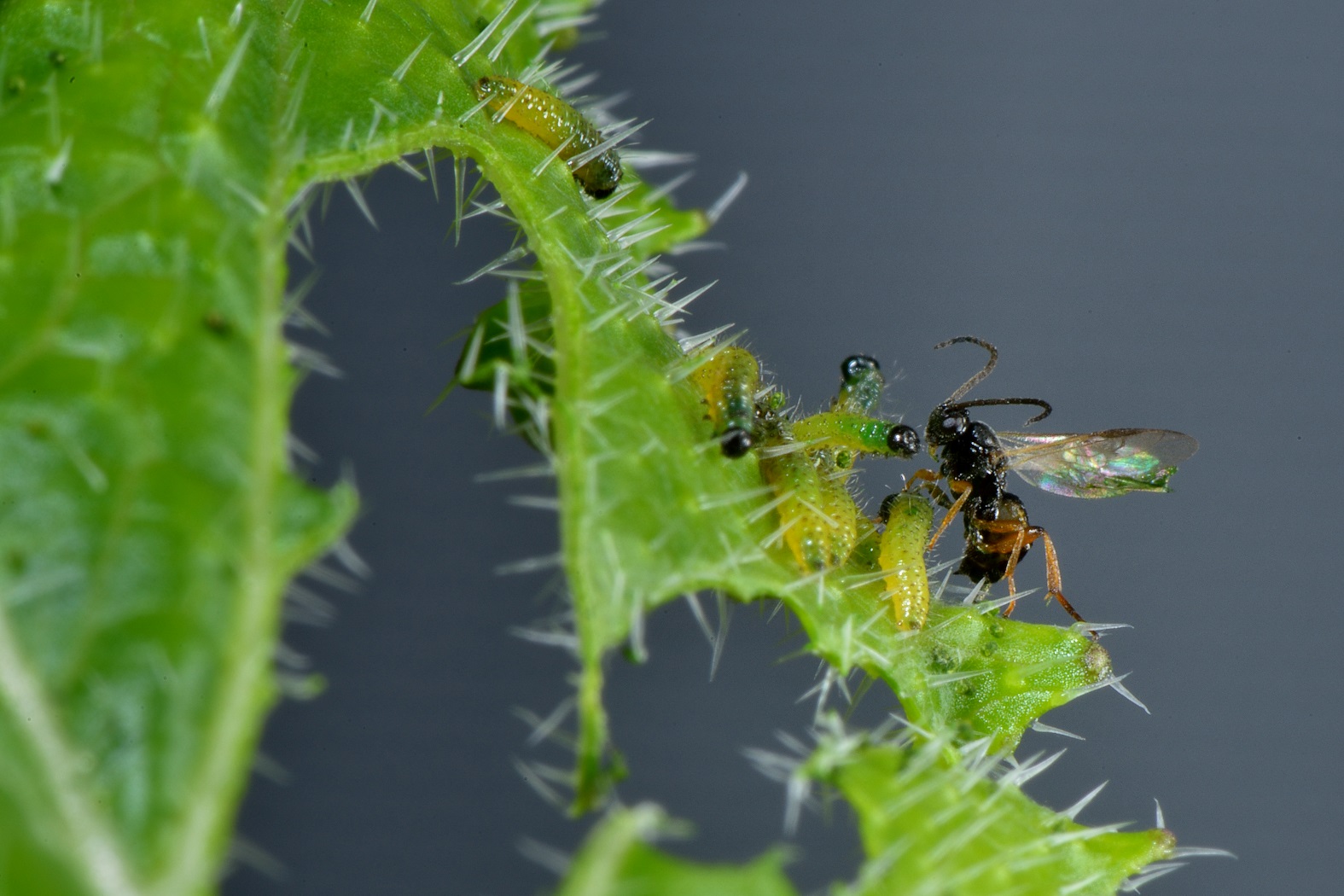


**Figure S1. *Cotesia glomerata* parasitoid attacking a group of young *Pieris brassicae* caterpillars on a *Brassica rapa* plant**. The wings of the parasitoid are stained by a droplet of spit regurgitated by one of the caterpillars under attack (green spot on the wings). As caterpillars grow larger, the volume of regurgitant they are able to produce increases. Photo: Neil VILLARD.


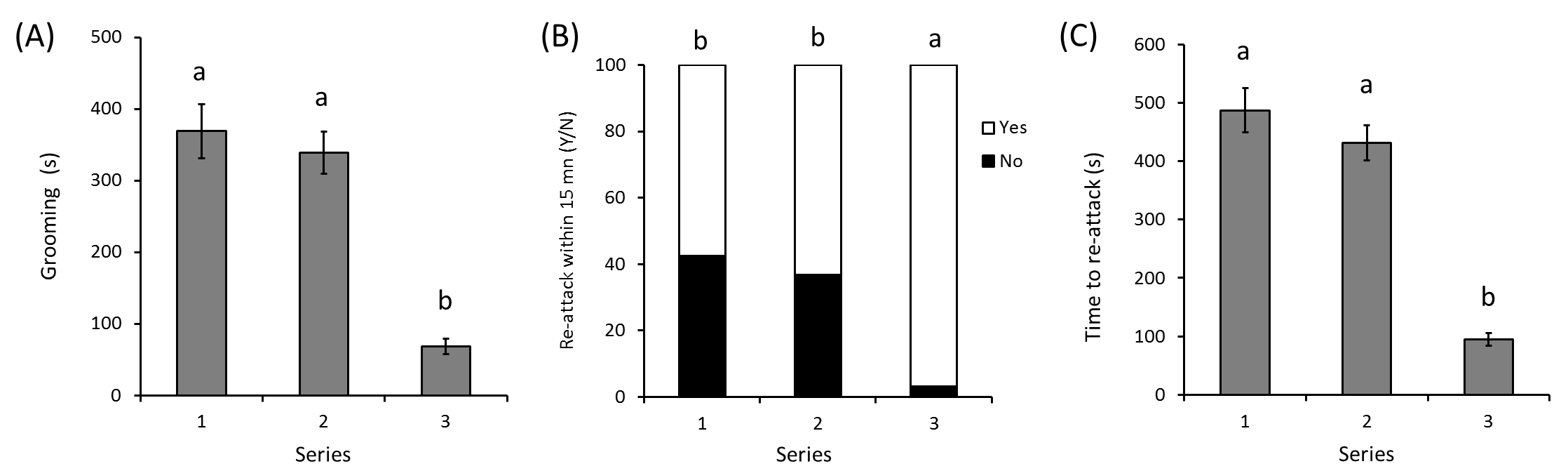


**Figure S2.** **Behavioral parameters of parasitoids after regurgitant exposure in three separate series.** (A) grooming time (seconds, mean + SE), (B) proportion of attack of a second caterpillar within 15 minutes (Y/N) after application of the regurgitant, (C) time from regurgitant exposure to the attack of a second caterpillar (seconds, mean + SE). Within each series, means followed by a different letter are statistically different (P < 0.05, Two-way ANOVAs for discrete variables, Chi-square tests for proportions, JMP9). Series 1: N = 92 observations; series 2: N = 144; series 3: N = 96.
